# Subclavian Artery Tortuosity Mimicking a Carotid Aneurysm: A Case Report

**DOI:** 10.7759/cureus.114299

**Published:** 2026-08-10

**Authors:** Rafael C Araújo

**Affiliations:** 1 Family Medicine, USF Dr. Pelaez Carones, Braga, PRT

**Keywords:** carotid artery aneurysm, case reports, color doppler ultrasonography, differential diagnoses, pulsatile neck masses, subclavian artery tortuosity

## Abstract

Pulsatile neck masses (PNMs) are relatively rare and require careful evaluation, as they have numerous potential differential diagnoses. A PNM may be of non-vascular or vascular origin, such as aneurysms and arterial tortuosities, which are anatomical variants. The distinction between these conditions should not be made solely based on physical examination. Arterial Doppler ultrasound is a useful method for the initial evaluation of PNM, allowing for the differentiation of aneurysms from vascular anatomical variants and thereby avoiding unnecessary interventions.

We describe the case of an 83-year-old independent female patient with a history of hypertension, atrial fibrillation, and dyslipidemia. During a follow-up visit, the patient reported a seven-month history of a pulsatile mass in the right anteroinferior cervical region. She noted that the mass became more prominent with elevated blood pressure and denied any other associated symptoms. On physical examination of the cervical region, no murmurs were heard on bilateral carotid artery auscultation; a pulsatile mass was present in the right anteroinferior cervical region, measuring approximately 3.5 × 2.5 cm. No other relevant findings were noted. A carotid artery aneurysm was considered in the differential diagnosis. A carotid and vertebral Doppler ultrasound was requested, which revealed that the mass corresponded to moderate tortuosity of the right subclavian artery, a benign anatomical variant. No therapeutic intervention was necessary, and the patient remains under regular clinical follow-up.

This case report demonstrates how a PNM caused by tortuosity of the right subclavian artery can be misinterpreted as a carotid artery aneurysm. Evaluation based solely on physical examination is insufficient, and arterial Doppler ultrasound is important for the initial assessment. The report underscores the importance of a thorough evaluation of PNMs and consideration of various differential diagnoses to avoid unnecessary procedures. This report contributes to the literature by highlighting that tortuosity of the right subclavian artery can present as a PNM and mimic a carotid artery aneurysm.

## Introduction

Although pulsatile neck masses (PNMs) are relatively rare, they warrant careful evaluation given their broad differential diagnosis. A PNM may be of vascular or non-vascular origin when it is closely related to the carotid artery or another cervical artery. Among the various vascular etiologies of PNM are aneurysms and arterial tortuosities, the latter of which represent anatomical variants that can involve multiple arterial territories, including the subclavian artery. These tortuous and redundant arteries can produce a large, pulsatile mass in the neck that is indistinguishable from an extracranial carotid artery aneurysm on physical examination. Although it has been suggested that this distinction can be made based on pulsatility, this is not recommended, since distinguishing between these conditions based solely on physical examination is difficult and imprecise, even if a thrill or murmur is present. Thus, arterial Doppler ultrasound is a useful method for the initial evaluation and differential diagnosis of PNM, allowing for the distinction between aneurysms and vascular anatomical variants and avoiding unnecessary interventions [1,2].

Although the prevalence of severe right subclavian artery tortuosity in the general population is unknown, studies have reported its presence in approximately 10% of patients undergoing cardiac catheterization procedures, including coronary angiography and transcatheter aortic valve implantation [3,4]. Right subclavian artery tortuosity is significantly more common and pronounced than that of the left subclavian artery. The literature suggests that this lateralized pattern of tortuosity may mirror similar findings in the internal carotid arteries, supporting a mechanical etiology. Age-related “aortic unfolding,” characterized by upward displacement, elongation, and counterclockwise rotation of the aortic arch, may contribute by increasing the curvature and mechanical stress on the right subclavian artery, which must accommodate a shortened anatomical course between its origin and the thoracic outlet [4].

The clinical predictors of right subclavian artery tortuosity vary slightly across different studies. One study identified hypertension, female sex, advanced age, non-smoker status, short stature, and a high BMI as predictors [3], while another identified only age ≥ 80 years, female sex, prior stroke, and thoracic kyphosis [4]. This latter study found no association between height and tortuosity, attributing this result to measurement bias associated with self-reported height, nor did it find an association between hypertension and tortuosity; the latter association remains controversial [4]. The various imaging modalities used to evaluate the vasculature of the upper extremities include ultrasound and color Doppler, CT angiography, magnetic resonance angiography, and digital subtraction angiography. Although there is sufficient evidence to consider CT angiography the test of choice for arterial evaluation of the upper extremities, digital subtraction angiography remains the gold standard for upper extremity vascular imaging because of its high resolution, as well as its therapeutic advantages, allowing diagnosis and treatment to be performed simultaneously [5].

Subclavian artery tortuosity is a recognized anatomical variant, and its identification relies on appropriate vascular imaging to distinguish it from other vascular abnormalities. This case report aims to describe the presentation and diagnostic evaluation of a PNM, while also discussing the possible differential diagnoses. This report was prepared in accordance with the CARE Guidelines [6].

This article was previously presented as an oral presentation at the “Horizontes em Medicina Geral e Familiar” lecture on November 27, 2025. Written informed consent was obtained from the patient for publication of this case report and the associated clinical data. Ethical approval was obtained from the Hospital de Braga Ethics Committee (approval no: 175_2025) in accordance with local ethical requirements.

## Case presentation

Patient information

The patient, an 83-year-old woman, attended a follow-up appointment for hypertension in February 2025. Toward the end of the visit, she reported a pulsatile mass in the right anteroinferior cervical region that had been present since July 2024. She reported that the mass became more prominent during episodes of elevated blood pressure. She denied headache, dizziness, visual disturbances, or other associated symptoms. Her past medical history included hypertension, permanent atrial fibrillation, dyslipidemia, hyponatremia, osteoporosis, and bilateral varicose vein surgery in 1990. There was no history of cerebrovascular accident or transient ischemic attack. Her long-term medication regimen consisted of candesartan, lercanidipine, simvastatin, apixaban, esomeprazole, lorazepam, bioflavonoids, and calcifediol. She had no known drug allergies. The patient did not smoke and did not consume alcohol or illicit substances. She was independent in activities of daily living and cognitively intact.

Clinical findings

**Figure 1:**
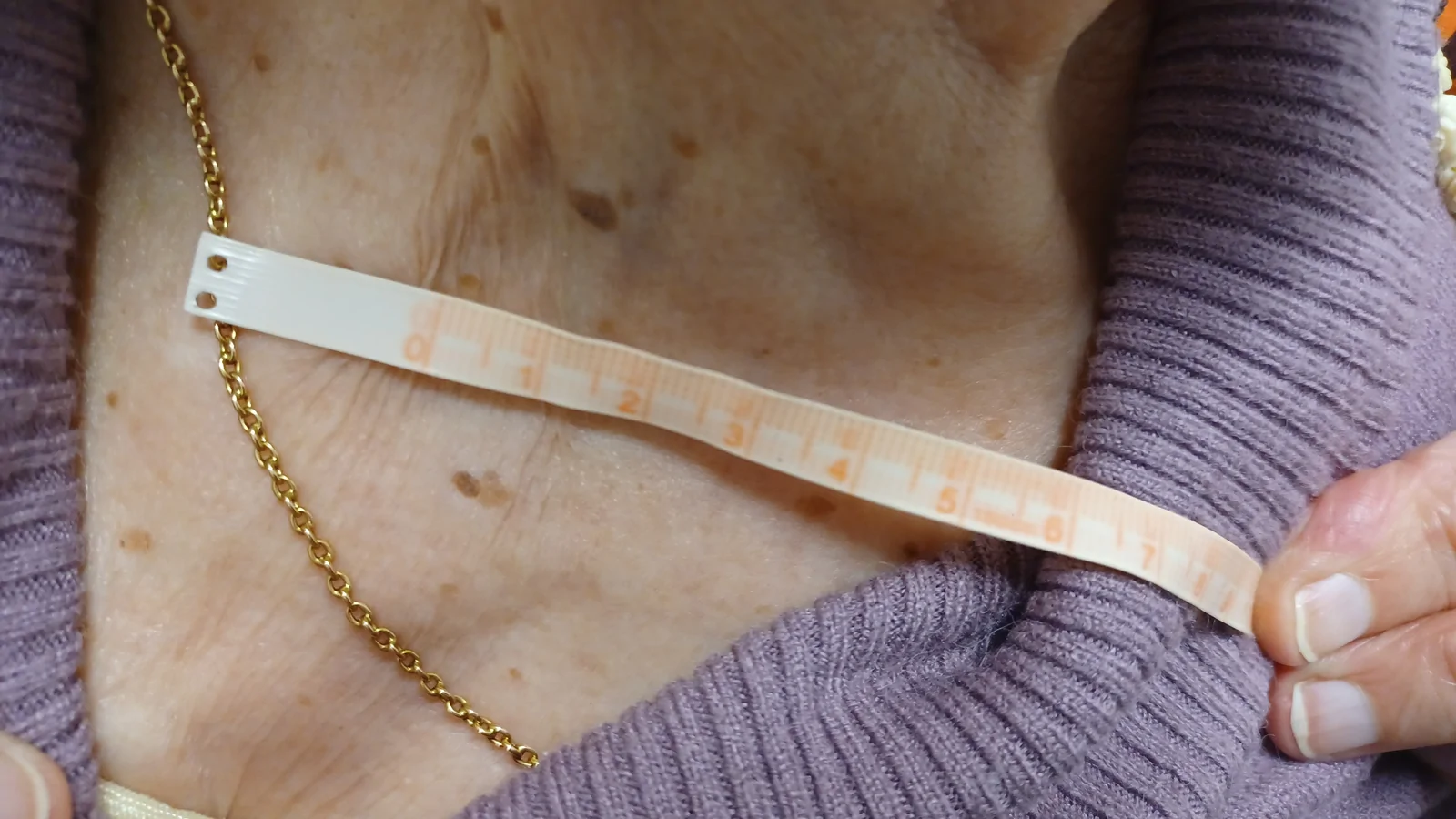
Pulsatile mass in the right anteroinferior cervical region, measuring approximately 3.5 x 2.5 cm

On initial physical examination, the patient was conscious, oriented, and cooperative, and was hemodynamically stable. Blood pressure measurements revealed values of 146/76 mmHg in the left upper extremity and 144/75 mmHg in the right upper extremity, with no clinically significant inter-arm difference. Heart rate was 63 bpm. Cardiac auscultation revealed an irregular rhythm and no murmurs. Pulmonary auscultation was unremarkable. Examination of the cervical region revealed a pulsatile mass in the right anteroinferior region, measuring approximately 3.5 × 2.5 cm (Figure 1). Carotid auscultation revealed no bruits bilaterally. The left carotid pulse was wide and irregular, consistent with atrial fibrillation. The remainder of the physical examination was unremarkable.

Timeline of tests and findings

**Figure 2:**
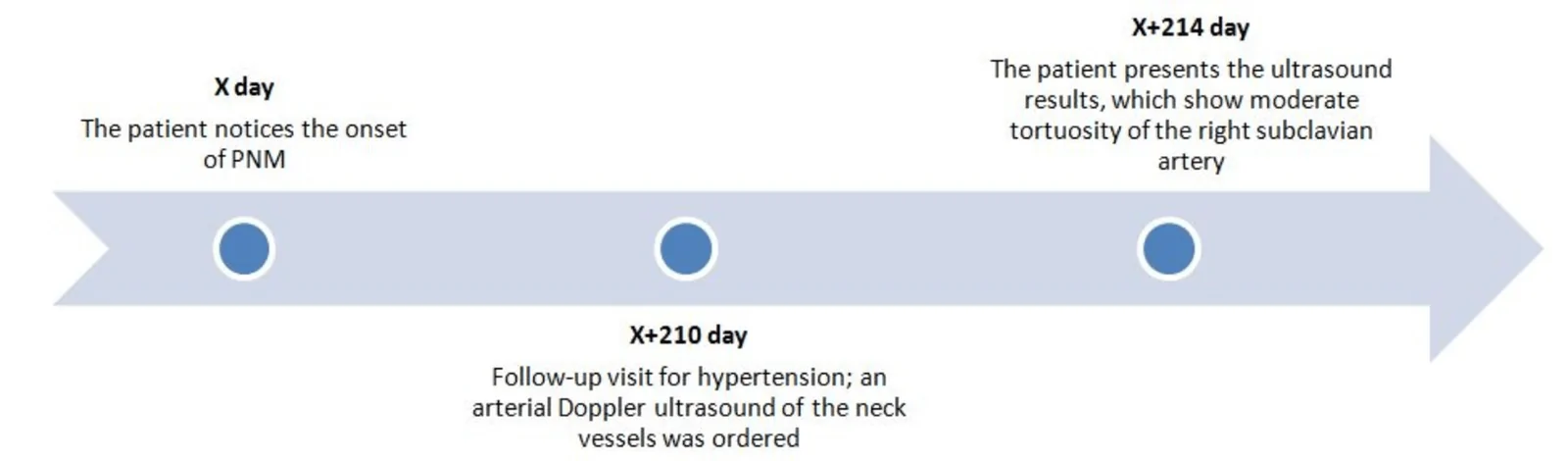
Timeline showing the most relevant tests and findings PNM: pulsatile neck mass

The timeline showing the most relevant tests and findings in this case is shown in Figure 2.

Diagnostic assessment

Initially, following a detailed medical history and physical examination, the primary differential diagnosis was a carotid artery aneurysm, due to the patient’s age, the location and pulsatility of the mass, its insidious onset, and history of hypertension and dyslipidemia. No laboratory tests were specifically requested for the evaluation of the suspected carotid artery aneurysm. However, selected routine laboratory investigations performed as part of the patient’s hypertension follow-up (Table 1) did not contribute to the diagnostic evaluation of the cervical mass.

**Table 1:** Selected routine laboratory results from hypertension follow-up Reference ranges and target values correspond to those provided by the performing laboratory HDL: high-density lipoprotein; LDL: low-density lipoprotein

Parameter	Result	Unit	Reference range/target value
Hemoglobin	13.6	g/dL	11.6-15.0
White blood cells	6.370	x10^9^/L	3.400-9.600
Platelets	311	x10^9^/L	157-371
Glucose	97	mg/dL	60-110
Creatinine	0.64	mg/dL	0.50-1.10
Total cholesterol	175	mg/dL	< 200
HDL cholesterol	88	mg/dL	> 50 (target for women)
LDL cholesterol	73	mg/dL	< 55 (target for very high cardiovascular risk)
Triglycerides	72	mg/dL	< 150

Previous routine cardiovascular assessment included electrocardiography and transthoracic echocardiography. Electrocardiography demonstrated atrial fibrillation with a controlled ventricular rate and complete left bundle branch block. Transthoracic echocardiography showed preserved biventricular systolic function (left ventricular ejection fraction of 71%), moderate biatrial enlargement, age-related mild degenerative valvular changes, mild-to-moderate mitral and tricuspid regurgitation, and no evidence of intracardiac masses or abnormalities of the thoracic great vessels. These examinations had been performed as part of routine follow-up for the patient’s pre-existing cardiovascular conditions and were not specifically requested for the evaluation of the cervical mass. The patient remained on regular follow-up for her known cardiovascular conditions, and no changes to her cardiovascular medication were required.

**Figure 3:**
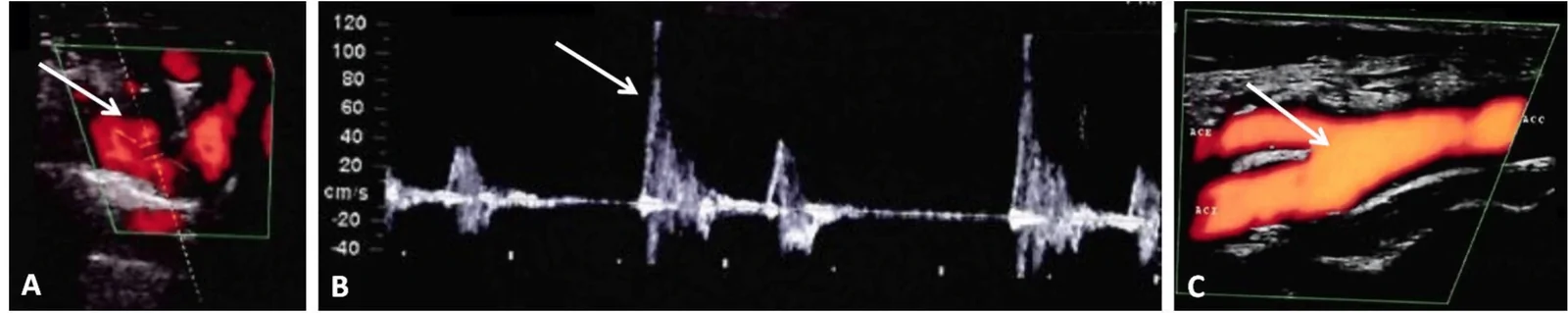
Arterial Doppler ultrasound of the neck vessels (A) Color Doppler image of the right subclavian artery (arrow), demonstrating preserved luminal patency with moderate tortuosity. (B) Spectral Doppler waveform (arrow) of the right subclavian artery showing normal flow velocities without hemodynamically significant changes. (C) Color Doppler image of the right carotid bifurcation (arrow) demonstrating preserved anatomy and flow within the common, internal, and external carotid arteries

The initial investigation consisted of an arterial Doppler ultrasound of the neck vessels, performed one week later (Figure 3), with the following findings:

Right Side

The examination showed preserved luminal patency of the brachiocephalic trunk, subclavian artery, and carotid axes, with moderate tortuosity of the subclavian artery, without significant luminal narrowing. Mild intima-media thickening and a homogeneous atheromatous plaque were present in the proximal portion of the internal carotid artery (length 5.6 mm; thickness 2 mm), with estimated stenosis of less than 50% (approximately 30%). Flow velocities and spectral characteristics were within normal limits. The vertebral artery demonstrated antegrade flow, without hemodynamically significant changes.

Left Side

The investigation revealed preserved luminal patency of the subclavian artery and carotid axes, with intima-media thickening and no evidence of atheromatous plaques or significant stenoses. Mild curvatures were observed in the middle/distal portion of the internal carotid artery. Flow was uniform, with a low-resistance diastolic pattern and no hemodynamically significant changes in the vertebral artery.

Conclusion

There were no significant morphological or hemodynamic changes in the carotid and vertebral arterial axes studied, bilaterally.

The absence of a focal vascular lesion or saccular outpouching on Doppler ultrasound, together with the lack of clinical features suggestive of a solid cervical mass on physical examination, made alternative causes of a PNM unlikely, including a carotid body tumor, pseudoaneurysm, jugular venous abnormalities, thyroid masses, and lymphadenopathy. The Doppler findings also argued against hemodynamically significant carotid artery disease and subclavian steal syndrome, as no relevant carotid or subclavian stenosis was identified and vertebral artery flow remained antegrade bilaterally. Based on these findings, the pulsatile mass was interpreted as being caused by tortuosity of the right subclavian artery, thus ruling out the possibility of a carotid artery aneurysm. As Doppler ultrasound was considered sufficient for the diagnosis by the vascular surgeon who performed the examination, no additional vascular imaging was recommended.

Therapeutic interventions, follow-up and results

The pulsatile mass was identified as being caused by tortuosity of the right subclavian artery, a benign anatomical variant. No therapeutic intervention was necessary. The patient was informed about the nature of the condition, and routine follow-up consisted of semiannual consultations for hypertension management. The patient has been followed for approximately 16 months (February 2025 to June 2026). Throughout this period, the pulsatile mass has remained clinically stable, with no changes in size or other characteristics. She remains asymptomatic and functionally independent. Due to the stability of the PNM and the absence of a clinical indication for further evaluation, no repeat Doppler ultrasound or additional vascular imaging was performed. No complications or need for intervention were identified. Blood pressure management followed standard hypertension guidelines, as no specific blood pressure targets are associated with this condition.

Patient perspective

The patient initially reported no prior knowledge of the PNM and expressed uncertainty regarding its nature. After diagnostic evaluation and explanation of the findings by the vascular surgeon and the attending physician, she appeared reassured by the benign diagnosis of a vascular anatomical variant. No formal patient-reported outcome measure was collected.

## Discussion

This report discusses the case of an 83-year-old woman who presented with a PNM, initially suggestive of a carotid artery aneurysm, which was subsequently identified as being caused by tortuosity of the right subclavian artery. This case highlights the importance of considering multiple differential diagnoses when encountering a PNM on physical examination. A limitation of this report is that it describes a single case; therefore, its findings should be interpreted with caution, as broader conclusions cannot be drawn without additional cases and further reports. Furthermore, advanced imaging modalities such as CT angiography, magnetic resonance angiography, as well as digital subtraction angiography, which is considered the gold standard for detailed vascular assessment, were not performed. Although Doppler ultrasound was considered sufficient for diagnosis by the evaluating vascular surgeon, and no additional vascular imaging was recommended given the absence of aneurysmal degeneration, significant stenosis, flow abnormalities, neurological symptoms, or upper limb ischemic symptoms, advanced imaging might have provided further anatomical characterization.

Although PNMs are relatively rare, the differential diagnoses associated with them are numerous. The etiology of these masses may be vascular or non-vascular, depending on whether they are closely related to an artery. Masses of vascular etiology include aneurysms (of the common carotid, brachiocephalic trunk, and right subclavian arteries) and arterial tortuosities (of the common carotid, brachiocephalic trunk, right subclavian, and external carotid arteries), which are anatomical variants; pseudoaneurysms of the common carotid artery; arteriovenous fistulas; carotid body tumors; a prominent or dilated carotid bulb; strong venous pulsations; and varicose veins of the right external jugular vein. Non-vascular masses include enlarged lymph nodes, neurogenic tumors, branchial cleft cysts, cystic hygromas, lipomas, thyroid tumors, aberrant thyroid tissue, and parathyroid cysts [1,2].

Due to their potential severity, carotid artery aneurysms are an important entity to consider in the differential diagnosis of a PNM. Carotid artery aneurysms are rare, accounting for only approximately 0.4% to 4% of all peripheral artery aneurysms [2,4]. Their main causes are atherosclerosis (34.2%), trauma (10%), and vasculitis (5.57%). The most common comorbidities associated with this condition are hypertension (13.9%), coronary artery disease (5.2%), and dyslipidemia (4.4%). The most common presenting symptom is a pulsatile mass in the neck (31.2%), followed by ischemia (24.7%), pain (9.75%), dizziness (8.3%), cranial nerve compression (8%), and central nervous system dysfunction (7.8%); patients may also be asymptomatic (5.7%) [7]. In this case, a carotid artery aneurysm was the initially suspected diagnosis, as the patient presented with a PNM and had a history of hypertension and dyslipidemia.

Both carotid artery aneurysms and right subclavian artery tortuosity may present as a PNM [1]. Doppler ultrasound is the preferred initial imaging modality for the evaluation of both entities. CT angiography and magnetic resonance angiography may be used for more detailed anatomical evaluation and confirmation. However, digital subtraction angiography remains the gold standard imaging modality for detailed vascular assessment. Correctly distinguishing between these two entities is important because their management differs considerably, thereby helping to avoid unnecessary interventions. While right subclavian artery tortuosity represents an anatomical variant and is usually managed conservatively in asymptomatic patients, carotid artery aneurysms may require surgical, endovascular, or hybrid intervention [5,7].

For the initial evaluation of a PNM, Doppler ultrasound is a particularly useful method, as it allows for real-time, non-invasive, and repeatable assessment of the mass, narrowing the differential diagnosis and guiding the initial investigation of the lesion. Although it has been suggested that the distinction between an aneurysm and tortuosity of the carotid artery can be made through palpation, this is not recommended, since it is difficult and imprecise to evaluate a PNM solely by physical examination, even in the presence of a thrill or murmur. Doppler ultrasound can determine whether a PNM corresponds to an aneurysm, establish the diagnosis of vascular masses, and further establish or strongly suggest the final diagnosis [1,2]. Thus, in the case presented, an arterial Doppler ultrasound of the neck vessels was requested.

In this case, the ultrasound revealed moderate tortuosity of the right subclavian artery, ruling out a carotid artery aneurysm. As previously described, right subclavian artery tortuosity is among the vascular causes of PNM [1]. The literature suggests that age-related “aortic unfolding” may contribute to the development of right subclavian artery tortuosity by increasing vessel curvature and mechanical stress, as the right subclavian artery must accommodate a relatively shortened anatomical course between its origin and the thoracic outlet [4]. This altered vascular anatomy may bring the artery closer to superficial cervical structures, allowing arterial pulsations to become clinically apparent.

Some studies have shown that severe tortuosity of the right subclavian artery is present in approximately 10% of patients undergoing cardiac catheterization procedures [3,4]. The clinical predictors of right subclavian artery tortuosity vary slightly across different studies. One study identified hypertension, female sex, advanced age, non-smoking status, short stature, and a high BMI as predictors [3], while another identified only age ≥ 80 years, female sex, prior stroke, and thoracic kyphosis [4]. Although the tortuosity was only moderate, the patient presented several of the described predictors: she was an 83-year-old woman, a non-smoker, of short stature (1.55 m), and with a BMI of 25.1 kg/m². No specific guidelines or published recommendations addressing the management of isolated asymptomatic subclavian artery tortuosity were identified. Therefore, management decisions should be individualized according to the clinical presentation and imaging findings. In the absence of symptoms, hemodynamic significance, or associated vascular complications, conservative management was considered appropriate in this patient.

## Conclusions

This case report demonstrates how a PNM caused by tortuosity of the right subclavian artery can be misinterpreted as a carotid artery aneurysm. Evaluation based solely on physical examination is insufficient in such cases, even in the presence of a thrill or murmur. Arterial Doppler ultrasound is a useful method for the initial evaluation of a PNM. This report highlights for clinicians the importance of thoroughly evaluating PNMs and considering a range of differential diagnoses in order to avoid unnecessary procedures. This report contributes to the literature by highlighting that tortuosity of the right subclavian artery can present as a PNM and mimic a carotid artery aneurysm.
